# Efficacy of Different Encapsulation Techniques on the Viability and Stability of Diverse Phage under Simulated Gastric Conditions

**DOI:** 10.3390/microorganisms11102389

**Published:** 2023-09-25

**Authors:** Sicelo B. Dlamini, Adriano M. Gigante, Steven P. T. Hooton, Robert J. Atterbury

**Affiliations:** 1 School of Agricultural Sciences, Faculty of Agriculture and Natural Sciences, University of Mpumalanga, Nelspruit 1200, South Africa; 2 School of Veterinary Medicine and Science, University of Nottingham, Sutton Bonington, Leicestershire LE12 5RD, UK; 3 Department of Genetics and Genome Biology, University of Leicester, University Road, Leicester LE1 7RH, UK

**Keywords:** bacteriophage, encapsulation, poultry, pig, monogastric, feed, biocontrol, microcapsules, antimicrobial resistance

## Abstract

*Salmonella* causes a range of diseases in humans and livestock of considerable public health and economic importance. Widespread antimicrobial use, particularly in intensively produced livestock (e.g., poultry and pigs) may contribute to the rise of multidrug-resistant *Salmonella* strains. Alternative treatments such as bacteriophages have shown promise when used to reduce the intestinal carriage of *Salmonella* in livestock. However, the digestive enzymes and low pH encountered in the monogastric GI tract can significantly reduce phage viability and impact therapeutic outcomes. This study deployed alginate–carrageenan microcapsules with and without CaCO_3_ to protect a genomically diverse set of five *Salmonella* bacteriophages from simulated gastrointestinal conditions. None of the unprotected phage could be recovered following exposure to pH < 3 for 10 min. Alginate–carrageenan encapsulation improved phage viability at pH 2–2.5 after exposure for 10 min, but not at pH 2 after 1 h. Including 1% (*w*/*v*) CaCO_3_ in the formulation further reduced phage loss to <0.5 log_10_ PFU/mL, even after 1 h at pH 2. In all cases, phage were efficiently released from the microcapsules following a shift to a neutral pH (7.5), simulating passage to the duodenum. In summary, alginate–carrageenan-CaCO_3_ encapsulation is a promising approach for targeted intestinal delivery of genomically diverse *Salmonella* bacteriophages.

## 1. Introduction

Combatting antimicrobial resistance (AMR) is one of the most pressing global challenges we face this century. An estimated 4.95 million deaths were associated with AMR worldwide in 2019, with 1.27 million of these directly attributed to AMR infections [1]. The effects of AMR pathogens are felt more keenly in low- and middle-income countries (LMIC) where health and sanitation systems may be inadequate the meet this challenge [2].

The use of antimicrobials in humans and livestock is often cited as a reason for the enrichment of antimicrobial resistance genes (ARG) in populations of both pathogenic and commensal bacteria [3]. The agricultural sector consumes approximately 70% of antimicrobials globally [4], with many countries still employing them as growth promoters. As global livestock production continues to expand and intensify, particularly in the pig and poultry sectors, antimicrobial usage is predicted to increase by up to 67% by 2030 in BRIC countries, where production is growing more rapidly than the global average [5].

Poultry and pork products remain central to the foodborne transmission of *Salmonella* globally [6]. *Salmonella* is also an important primary pathogen in both poultry and pigs, leading to morbidity and mortality, abattoir rejections, and subsequent economic loss for the farmer [7]. AMR in *Salmonella* has increased significantly from the 1960s in the USA and EU, particularly to older classes of antibiotics such as the tetracyclines, sulfonamides, ampicillin, and streptomycin [8].

The most recent EU surveillance data show that a higher proportion of multidrug-resistant (MDR) *Salmonella* can be isolated from broiler chicken carcasses (51.2%) than humans (22.6%) in this region [9]. However, these summary data mask an uneven distribution of AMR between serotypes, with higher levels of AMR recorded for *S*. Kentucky (54.8%) and monophasic *S.* Typhimurium (78.4%) compared with *S.* Enteritidis (1.9%). The recent emergence of *Salmonella* Infantis in broiler chickens carrying a megaplasmid conferring resistance to multiple antimicrobials as well as biocides and heavy metals has caused great concern [10]. Over 70% of these isolates are MDR, with many exhibiting exceptionally high resistance to third-generation cephalosporins [8]. Moreover, recent isolates of pESI-carrying *S*. Infantis strains from Italy were also found to be resistant to colistin [11], a Critically Important Antibiotic (CIA) and last resort for the treatment of pan-resistant bacterial infections [12]. Alternatives to antimicrobials for the control of *Salmonella* in poultry and pigs are urgently needed.

Bacteriophage (phage) and their derivatives have been mooted as among the most promising alternatives to antibiotics [13]. In addition to their potential in controlling bacterial diseases, dietary supplementation with bacteriophage has been associated with improvement in growth performance, gut microbiome, and production traits in broiler chickens [14,15], although this has been disputed [16]. However, questions remain about the optimisation of oral phage delivery in livestock, given that the stability and activity of phage across different pH, temperature, and physicochemical environments can vary considerably [17,18]. The low pH of the stomach in pigs (and proventriculus/gizzard in poultry) is an effective barrier which protects the small intestine from pathogen incursions [19]. Gastric pH varies across monogastric species, and also with age/stage of development, but is usually in the range of 2.6–4.2 [20], which is beyond the tolerance of many tailed phage, which are typically stable between a pH of 5 and 9 [17].

The particular challenge of delivering phage across the gastric acid barrier while maintaining a stable titre has been mitigated by oral administration of antacids (e.g., calcium carbonate or bicarbonate), and microencapsualtion in protective polymers [21,22]. The function of microencapsulation is both to protect phage from adverse environmental conditions and also to control the site at which the phage are released [23,24]. Additionally, encapsulation improves phage shelf life during storage and ensures constant release at the target site [25,26]. Although microencapsulated phage are more stable in acidic conditions compared to free phage, little is known about the effect of encapsulation on individual phages administered as a cocktail in the gastrointestinal environment [21]. One of the most used gel-forming agents for phage encapsulation is sodium alginate which has the advantage of allowing encapsulation under very mild conditions, crosslinking with calcium ions [18]. Other gelling agents have been tested in combination with sodium alginate to achieve improved performance (e.g., increase encapsulation efficiency), such as κ-carrageenan [27,28].

In vitro and in vivo studies have demonstrated the importance of using phage cocktails rather than single-phage preparations to achieve better therapeutic outcomes and reduce the emergence of phage resistance [29,30]. However, genetically diverse phage may differ in their tolerance of chemical and physical stresses encountered during microencapsulation [18], or be protected or released less effectively. This may result in less predictable phage therapy outcomes if only a subset of the phage in the cocktail are able to replicate successfully. In turn this may influence selection and modelling of phage at the in vitro stage [31].

The aim of the present study was to assess the viability and stability of five genetically diverse *Salmonella* phage encapsulated in alginate and carrageenan/calcium carbonate microcapsules under simulated gastrointestinal conditions.

## 2. Materials and Methods

### 2.1. Bacteria and Bacteriophage

Phage RA40, RA112, RA124, RA140, and RA148 were isolated from human sewage using host strain *Salmonella* Typhimurium 4/74 [32] using the method described by Van Twest and Kropinski [33]. Phage isolates were serially plaque-purified a minimum of three times before propagation and concentration (described below). Titration of phage stocks was performed using double-agar overlays.

### 2.2. Phage Propagation and Concentration

All phage stocks were propagated in 100 mL of Luria Bertani (LB) medium (Sigma-Aldrich/Merck, Darmstadt, HE, Germany) in a 250 mL/L screw-cap Erlenmeyer flask. The pre-warmed medium was inoculated with 100 µL of a mid-exponential culture of *S.* Typhimurium 4/74 (0.5 OD_600_). This culture was then incubated at 37 °C, shaking at 150 rpm until reaching 0.5 OD_600_. This culture was infected with 0.5 mL of a 10^6^ PFU/mL phage stock to achieve a multiplicity of infection (MOI) of 0.01. Incubation of the flasks was continued for up to 12 h, or until lysis was observed. Following incubation, 20 µL of chloroform was added to the lysate, which was then centrifuged at 4000× *g* for 10 min at 4 °C. The pellet was discarded, and the supernatant used for polyethylene glycol (PEG) concentration [34]. Briefly, DNase I (Roche Diagnostics, Mannheim, Germany) and RNase (Roche Diagnostics, Mannheim, Germany) were added to 30 mL of filtered (0.22 µm) lysate to a final concentration of 1 µg/mL and incubated for 30 min at room temperature. Subsequently, PEG-8000 (10% *w*/*v*) and NaCl (final concentration 1 M) were added and mixed at room temperature until fully dissolved. The suspension was then incubated at 4 °C overnight to allow the phage to precipitate. The suspension was then centrifuged at 11,000× *g* for 10 min at 4 °C to pellet the phage. The resulting supernatant was discarded, and the pellet was soaked in 2 mL SM buffer (50 mM Tris-HCl pH 7.5, 100 mM NaCl, 8 mM MgSO_4_) for 1 h at room temperature. An equal volume of chloroform was added to the suspension before being mixed gently and centrifuged at 3000× *g* for 15 min at 4 °C. The aqueous phase, containing the phage, was then collected and stored at 4 °C until further use.

### 2.3. Bacteriophage DNA Genome Extraction

High titre stocks (>10^9^ PFU/mL in SM buffer) of each phage were used to obtain genomic DNA preparations for sequencing. For degradation of phage particles, proteinase K was added to each preparation (100 μg/mL) prior to incubation at 55 °C for 30 min. For the extraction of phage genomic DNA each sample was subjected to a modified DNA Wizard (Promega, Southampton, UK) extraction procedure as described previously [35]. Briefly, 1 mL of DNA binding resin was added to each sample and the mixture was inverted gently several times. A wide-bored pipette was used to transfer the mixture into 3 mL syringe barrels attached to DNA wizard mini-columns. Plungers were reattached to the syringes and the samples were slowly passed through the mini-columns and the flow-through was discarded. To each syringe barrel 2 mL of 80% (*v*/*v*) isopropanol was added and passed through the columns as described above. Each mini-column was transferred to clean 1.5 mL tubes and centrifuged at 13,000× *g* for 2 min. For elution of DNA, molecular biology-grade water heated to 80 °C was added to each column prior to centrifugation at 13,000× *g* for 1 min. The DNA yield of each sample was quantified using Qubit dsDNA assay (Thermo Scientific, Swindon, UK) prior to whole genome sequencing.

### 2.4. DNA Sequencing & Assembly

Phage genomic DNA was sequenced using the Illumina NextSeq 2000 platform operating to produce 2 × 150 bp reads (Seq-Centre, Pittsburgh, PA, USA). Briefly, DNA libraries were sequenced from RA40, RA112, RA124, RA140, and RA148 using the Illumina DNA Prep Kit with the addition of IDT 10 bp UDI indices (Illumina, San Diego, CA, USA) generating 3.3 M–4.9 M reads per sample. Post-sequencing quality control (QC), adapter trimming, and demultiplexing of samples was performed using bcl-convert (v3.9.3). Following QC and trimming, FastQ files from each paired-end library were subsampled using the Randomly Subsample Reads—v1.0.2 function in KBase [36] to achieve approximately 30× coverage of each phage genome. Subsampled read-sets were assembled using SPAdes v3.15.3 [37] with this process being repeated iteratively if necessary to obtain the desired sequencing coverage. FastA nucleotide sequences for each phage were annotated using PROKKA and deposited in NCBI/Genbank. BLASTN analysis of each nucleotide sequence was performed to assign each phage to the correct family. The assembled genomes were screened for the presence of antimicrobial resistance and virulence genes using the CARD and VFDB databases, respectively [38,39]. The lifecycle of each phage (lytic, temperate) and DNA packaging were determined using PhaBOX [40], and PhageTerm [41], respectively.

### 2.5. Phage Phylogenetic Analysis

The complete nucleotide sequences of phages RA40, RA112, RA124, RA140, and RA148 were subjected to BlastN analysis allowing for identification of homologous genomes and phage families. From this initial screen, 50 genomes for each phage genus (*Jerseyvirus*, *Epseptimavirus*, and *Kuttervirus*) were downloaded in FastA format, and incorporated into a single file. A proteomic tree was constructed using the VIPTree tBLASTx algorithm allowing classification of all five RA phages in their respective genera [42]. Analysis of genetic relatedness based on genome-wide sequence similarities in the dataset was used to construct a proteomic tree.

### 2.6. Encapsulation of Phage in Alginate–Carrageenan (ALG-CG)

All phage stocks were diluted to 10^8^ PFU/mL in SM buffer prior to encapsulation. To produce the microcapsules, small-volume (10 mL) batches were formulated by extrusion of sodium alginate and κ-carrageenan (both from Sigma-Aldrich/Merck, Darmstadt, HE, Germany) as described previously [27], with some modifications. The sodium alginate stock solution was dissolved in distilled water to a concentration of 5% (*w*/*v*) and sterilised at 121 °C for 15 min. The κ-carrageenan stock solution was prepared by dissolution in hot (60 °C) sterile distilled water to 0.8% (*w*/*v*) final concentration.

Phage stock (1 mL, 10^8^ PFU) was added to 9 mL alginate–carrageenan (ALG-CG) mixture (2:0.3 *w*/*w* ratio) and stirred at room temperature for up to 2 min until visibly homogenised. For the extrusion method, a 22 G needle was used to eject the phage-ALG-CG solution, dropwise into a beaker containing 200 mL crosslinking solution (sterilised solution of CaCl_2_ 2% *w*/*v* dissolved in water) at approximately 200 rpm using a magnetic stirrer. After standing for 30 min in the crosslinking solution, the microcapsules were collected by decanting into a 50 mL falcon tube (Greiner Bio-One GmbH, Frickenhausen, Germany) and washed three times with 10 mL of sterile distilled water. Excess water on the surface of the microcapsules was capillary absorbed using a kimwipe. ALG-CG-CA microcapsules were prepared as above but to the ALG-CG mixture a suspension of powdered CaCO_3_ (Sigma-Aldrich/Merck, Darmstadt, HE, Germany) in deionised water was added to obtain a ALG:CG:CA ratio of 2:0.3:1 (*w*/*w*).

### 2.7. Encapsulation Efficiency and Particle Size Measurement

The phage encapsulation efficiency (EE) was determined in order to evaluate the number of phage that are effectively inside the microcapsules compared to the total amount of phage used on the formulation, using the following equation, EE(%) = (Pr/Pt) × 100, where Pt (PFU/mL) = phage titre added to the microencapsulation formulation mixture and Pr (PFU/mL) = phage titre released from the microcapsules [27]. Pr was determined using the double-agar layer method after dissolving microcapsules (1 g) for 20 min in 9 mL of dissolution solution (50 mM sodium citrate, 200 mM sodium hydrogen carbonate, and 50 mM Tris-HCl pH 7.5) [43]. The titre of free phage in the used crosslinking solution after encapsulation was determined using double-agar overlays.

Particle dimensions were determined by selecting a random sample of 30 microcapsules on a Petri dish using a ChemiDoc (Bio-Rad Europe GmbH, Basel, Switzerland) for image acquisition and ImageJ for measurement (U. S. National Institutes of Health, Bethesda, MD, USA). Statistical significance between ALG-CG and ALG-CG-CA groups was determined using an unpaired two-tailed *t*-test.

### 2.8. Susceptibility of Free and Microencapsulated Phage to Simulated Gastric and Duodenum Conditions

Phage in free form (non-formulated) were tested at specific pH conditions to determine their susceptibility to neutral or acidic pH conditions over time. Briefly, 100 µL of phage suspension at 1 × 10^6^ PFU/mL was added to 900 µL of defined pH solution (adjusted using concentrated HCl 37% with 5% NaCl (*w*/*v*), Sigma-Aldrich/Merck, Darmstadt, HE, Germany) at pH = 7.5 (control), 5, 4, 3, 2.5, and 2. After 10 min and 60 min incubation at 37 °C the phage titre was determined using double-agar overlays.

Phage susceptibility to simulated gastric conditions was determined as described previously [44], with some adaptations. Briefly, for simulated gastric fluid (SGF), freshly prepared wet microcapsules containing the phage (1 g) were placed in 9 mL (pH 2.0, 3.2 mg/mL pepsin, Sigma-Aldrich/Merck, Darmstadt, HE, Germany, 0.5% NaCl) and then incubated at 37 °C, 150 rpm for 10 min, 30 min or 1 h. They were then separated by decanting and washed three times with sterile water. The microcapsules were then allowed to disintegrate by placing them into 10 mL SM buffer and incubated at 37 °C, 150 rpm for 1 h. The titre of released phage was determined using the double-agar overlay plaque assays. For simulated intestinal fluid (SIF) the medium used was 9 mL of 0.5% NaCl water solution, pH 7.0 supplemented with 10 mg/mL pancreatin (Sigma-Aldrich/Merck, Darmstadt, HE, Germany), to which 1 g microcapsules containing the phage were added and then incubated at 37 °C, 150 rpm for 1 h. After incubation, and the visible dissolution of the particles, phage titration was performed by double-agar overlay.

### 2.9. Statistical Analysis

Phage numbers recovered from each experimental group were log_10_-transformed, before calculating summary statistics (mean, standard error). The significance of differences between groups was determined using a two-way analysis of variance (ANOVA). Differences between recovery of individual encapsulated and unencapsulated phage incubated under different pH conditions were determined using the Mann–Whitney U test. All analyses were performed using R v4.3.1 [45] with graphs plotted with ggplot2 v3.4.3 [46].

## 3. Results

### 3.1. Phage Genome Characteristics

A summary of the DNA sequencing and assembly for each phage, along with their accession numbers, is presented in Table 1. The five phage isolated in this study were all tailed, containing linear double-stranded DNA genomes. All five phage were predicted to be lytic, and contained no known antimicrobial resistance or virulence genes. BlastN analysis of each genome allowed each phage to be grouped within the genera of *Epseptimavirus* (RA40), *Jerseyvirus* (RA112, RA124, and RA140), and *Kuttervirus* (RA148). Phage RA148 was predicted to package its DNA using a headful (or pac) system. No prediction could be made for the remaining phage, although all of them showed evidence of circularly permuted genomes, which is consistent with the previous reports of Kutterviruses and Jerseyviruses [47].

**Figure 1:**
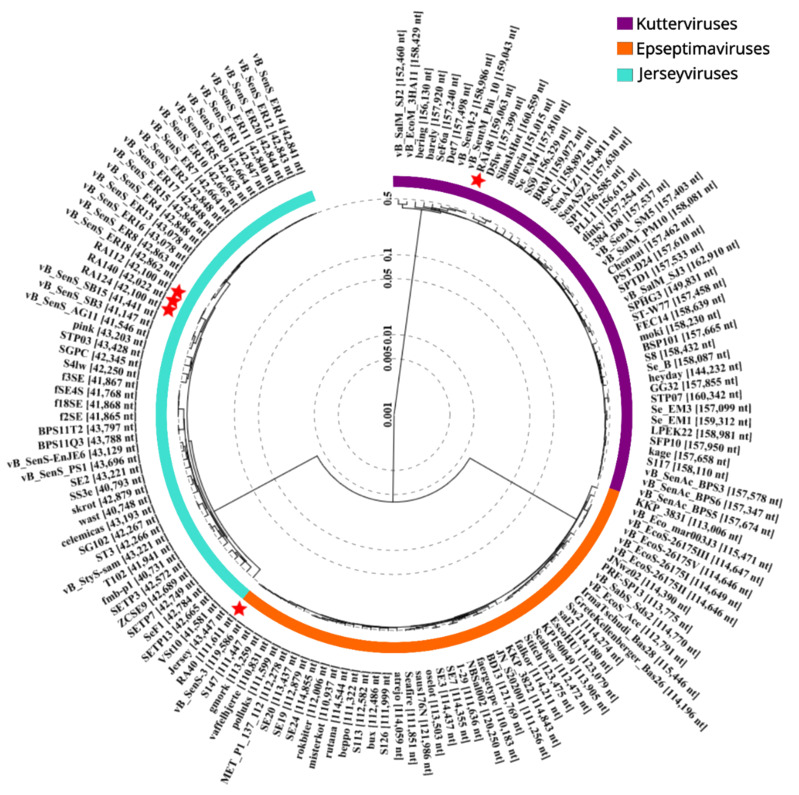
Proteomic phylogenetic tree. Circular proteomic tree generated by VIPtree including the five Salmonella phages represented in this study labelled with red stars.

In order to assess the relationships between the RA and related phages, a proteomic tree was constructed from fifty closely related phage genomes for each family using VIPtree [42]. Information regarding average nucleotide identities (ANI) across total genome length (%) are also included. Figure 1 shows the relatedness of each RA phage isolate (designated red stars) to other phage genome sequences. Each phage family is highlighted with separate colours for *Kuttervirus* (purple), *Epseptimavirus* (orange), and *Jerseyvirus* (turquoise).

*Epseptimavirus* RA40 is observed to occupy a branch with several other phages from the same family including vB_SenS_3 (MT004791), S147 (NC_048012), and gmork (MT074463). vB_SenS_3 has an ANI of 98.9% over 98.4% of the total genome length of RA40, while S147 has an ANI of 98.6% over 99.3%. The highly similar *Jerseyvirus* phages RA112, RA124, and RA140 occupy a distinct branch on the proteomic tree and are observed to be highly related to several sequences designated vB_SenS_SB3 (NC_073213) vB_SenS_SB15 (MK759883), and vB_SenS_AG11 (NC_041991). vB_SenS_SB3 has an ANI of 93.9% across 92.2% of the total genome lengths of RA112, RA124, and RA140; similar results are observed for vB_SenS_SB15 (93.8% ANI over 92.2% genome length). Kuttervirus RA148 is observed to be highly related at the nucleotide level (ANI 99.9–100% across total length of RA148 genome) to phages vB_SenM_2 (KX171211) and vB_SentM_Phi10 (MN384979).

### 3.2. Encapsulation Efficiency

Free (unprotected) phage are unlikely to remain viable in significant numbers after passage through the gastric acid barrier of pigs or poultry; therefore, microencapsulation formulations were tested to protect the phage from these conditions. The objectives were to provide protection from low pH and enzymatic digestion, obtain a high encapsulation efficiency and achieve efficient phage release. Two formulations were tested: sodium alginate and κ-carrageenan (ALG-CG) a well-known composite natural polysaccharide vehicle used for oral drug delivery systems [28], and ALG-CG-calcium carbonate (ALG-CG-CA) where the carbonate is added to provide enhanced pH buffering capacity within the microcapsule environment [48].

A summary of the encapsulation efficiency of each phage in both formulations is presented in Table 2. All five phage were encapsulated with high efficiency (>95%), with both formulations. The presence of calcium in the formulation did not impact the EE% (*p* > 0.28). Titration of the used crosslinking solution showed that, on average, less than 0.5% of the total amount of dispensed phage could be recovered in free form, which is consistent with high EE% recorded, and also suggests minimal dissociation of phage from the capsules over the 30 min crosslinking incubation time.

Mean particle size measurements (MPS) show that ALG-CG particles are significantly larger than ALG-CG-CA (*p* ≤ 0.01) which may be the result of calcium carbonate density promoting a smaller droplet size when extruding through the needle, consequently allowing for smaller particles to be formed.

### 3.3. Viability of Unencapsulated Phage at Different pHs

**Figure 2:**
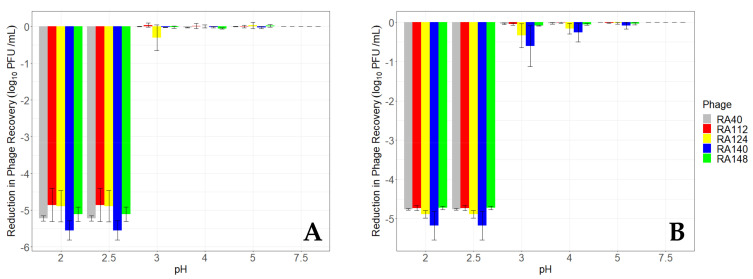
Recovery of unencapsulated (free) phage after incubation at different pHs. Phage viability, measured as the ability to form plaques on agar overlays, was determined after incubation at each pH for 10 min (**A**) and 60 min (**B**). The mean reduction in recovery (log_10_ PFU/mL ± S.E.) relative to the initial inoculum (~5 log_10_ PFU/mL) is presented for each phage, based on three biological replicates.

The recovery of unencapsulated (free) phage following incubation for 10 and 60 min in a range of pHs is presented in Figure 2. In all cases, the recovery of phage fell below detectable levels (10 PFU/mL) after 10 min incubation at pH 2.5 or below, from an initial inoculum of ~5 log_10_ PFU/mL. More variability in the recovery of individual phage was seen after 10 min than 60 min. Reductions in phage RA140 (blue) were greater at pH 2 and 2.5 (5.5 log_10_ PFU/mL) than all other phage after 10 min incubation. This was approximately 0.68 log_10_ PFU/mL greater than phage RA112, which was the least susceptible to low pH, although this difference was not significant (*p* = 0.72). Extending the incubation time to 60 min resulted in a similar, but more uniform, pattern of reduction at pH 2 and 2.5, with phage RA140 remaining the most susceptible to these pH conditions. This phage also appeared more susceptible than other phage between pH 3–5, with reductions of between 0.6 and 0.09 log_10_ PFU/mL, respectively, compared with the least susceptible phage, RA40, which was reduced by 0.04 to 0.002 log_10_ PFU/mL, respectively. However, differences in the recovery of phage after 60 min incubation at different pHs were not significant (*p* = 0.46).

### 3.4. ALG-CG Microcapsules Can Protect Phage from a Simulated Gastric Environment

**Figure 3:**
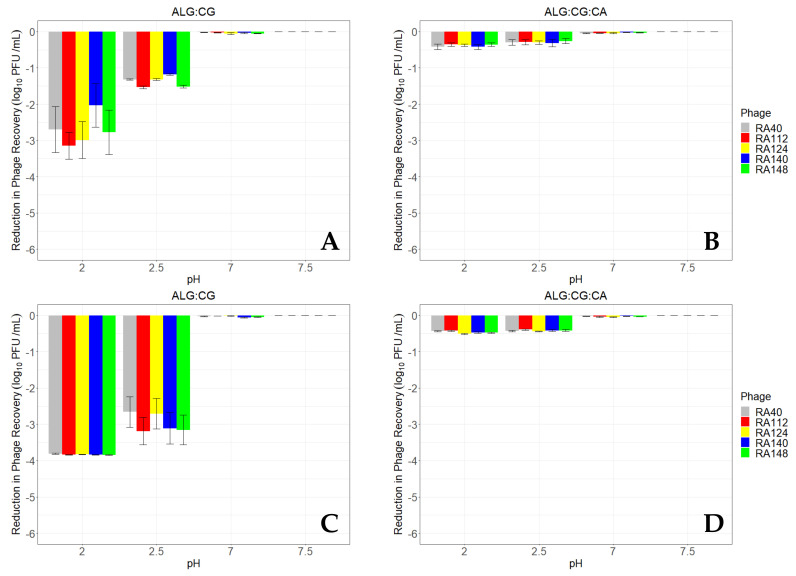
Recovery of encapsulated phage after incubation at different pHs. Mean reduction in phage recovery in ALG-CG microcapsules after exposure to each set of pH conditions for 10 minutes (**A**) or 60 minutes (**C**), and phage mean reduction in recovery in ALG-CG-CA microcapsules after 10 minutes (**B**) or 60 minutes (**D**) as determined by plaque formation on agar overlays. Simulated gastric fluid conditions were simulated by supplementing pH 2 and pH 2.5 solutions with pepsin. pH 7 was used to simulate intestinal fluid (SIF) and pancreatin was added. pH 7.5 was used as control, using SM buffer without enzyme supplementation. The mean reduction in recovery (log_10_ PFU/mL ± S.E.) relative to the initial inoculum (~4.8 log_10_ PFU/mL) is presented for each phage, based on four biological replicates.

The viability of phage encapsulated in sodium alginate and κ-carrageenan, with and without CaCO_3_, following incubation for up to 60 min under various pH conditions is presented in Figure 3. The phage encapsulated using AL-CG were subjected to different pH and enzymes to simulate the monogastric GIT environment in vitro. When subjected to control conditions (pH 7.5, without enzymes) each phage was released from the microcapsules at similar levels (mean 4.8 log_10_ PFU/mL) irrespective of the incubation duration. This is to be expected as the EE% was similar for all the phage tested here. The number of phage recovered following incubation for up to 60 min in SIF was similar to that recorded for pH 7.5 suggesting that exposure to pancreatin did not impact phage viability significantly.

Phage recovery from ALG-CG encapsulated phage following incubation at pH 2 and pH 2.5 offered some protection compared with unencapsulated phage. After 10 min incubation, phage recovery reduced by 2.03 to 3.14 log_10_ PFU/mL at pH 2, compared with 4.86 to 5.55 log_10_ PFU/mL for unencapsulated phage. The protective effect of ALG-CG encapsulation was more evident at pH 2.5, where mean reductions in phage after 10 min incubation were 1.32 to 1.53 log_10_ PFU/mL, compared with 4.56–5.55 log_10_ PFU/mL for unencapsulated phage.

Extending the incubation of ALG-CG encapsulated phage to 60 min demonstrated that phage recovery was higher than for unencapsulated phage, but that protection was incomplete. Phage recovery after incubation at pH 2 was reduced by 3.81 to 3.85 log_10_ PFU/mL compared with 4.73 to 5.18 log_10_ PFU/mL for unencapsulated controls. Similarly to the 10 min incubation at pH 2.5, encapsulation was proportionately more effective compared with the unencapsulated controls after 60 min incubation. Reductions in the recovery of encapsulated phage ranged from 2.66 to 3.19 log_10_ PFU/mL compared with 4.73 to 5.18 log_10_ PFU/mL for unencapsulated controls. Recovery of phage after incubation at pH 7 and 7.5 was relatively unchanged (reductions ranged between 0 and 0.05) regardless of incubation time.

The addition of CaCO_3_ to the encapsulation formula was associated with improved recovery for all five phage, following incubation under simulated gastric conditions for both 10 and 60 min. Protection of phage at pH 2 and 2.5 after both incubation times was similar, with reductions of between 0.35 to 0.42 log_10_ PFU/mL (10 min) and 0.39 to 0.51 log_10_ PFU/mL (60 min). ALG-CG-CA encapsulated phage recovery was significantly higher than for those encapsulated in ALG-CG alone, or which were unencapsulated (*p* < 0.05). These phage were also protected more uniformly, with no significant differences between the recovery of individual phage within each pH tested (*p* > 0.84).

## 4. Discussion

Previous studies have tested phage encapsulation from the exclusive perspective of pharmaceutical delivery and vehicle optimisation [27,43,49]. However, the effect of encapsulation on the protection and release of genomically distinct phage is poorly understood. This is an important aspect to consider when designing therapeutic preparations, as these are typically delivered as a cocktail of different phage.

The phages used in this study belong to three distinct phage genera, *Jerseyvirus*, *Epsptimavirus*, and *Kuttervirus*, representing the Guernseyvirinae subfamily and the Ackermannviridae and Demerecviridae families, respectively. Phages RA112, RA124, and RA140 represent a distinct subspecies in the Jerseyviruses occupying a unique branch in the proteomic tree. The primary host-encoded receptor for Jerseyviruses is reported to be the *Salmonella* O-antigen/lipopolysaccharide (LPS) [50]. Multiple studies have highlighted the desirable antimicrobial properties of Jerseyviruses. Rapid adsorption to host cells, short latent periods, high burst sizes, and broad host-ranges against a wide range of *Salmonella* serovars are hallmark features [51,52,53]. Phage RA40 constitutes a member of the *Epseptimavirus* phage family which is also well-noted for potential biocontrol applications of *Salmonella* spp. For instance, *Epseptimavirus* STG2 was found to be proficient in reducing *S*. Enteritidis and *S*. Typhimurium in planktonic biofilms formed on biotic (cabbage) and abiotic (polystyrene and stainless steel) surfaces [54]. Epseptimaviruses have genomes ranging from 110–125 kb in length with ~40% GC content (40–111,611 bp–39.99% GC). The host-encoded receptor targeted by Epseptimaviruses is outer membrane protein BtuB (vit B12 receptor) [55]. BtuB is highly conserved throughout *Enterobacteriaceae* and *Epseptimavirus* ESCOHU1 is reported as being capable of infecting *Salmonella* spp. and *E. coli* O157:H7 [56]. Phage RA148 was determined to be a member of the broad host-range, *Enterobacteriaceae*-infecting *Kuttervirus*. The *Kuttervirus* phage genus has a diverse host-range with numerous members noted for their capacity to target significant human and animal pathogens including *E. coli*, *Klebsiella pneumoniae*, and *Salmonella* spp. *Kuttervirus* genomes are quite variable in length and are observed to range from 144 kb–164 kb with 44–45% GC content (148–159,063 bp–44.71% GC). Kutterviruses are recognised as encoding three to four separate tail spike proteins and for those infecting *Salmonella*, the O-antigen is the primary receptor [57,58,59,60].

The present study demonstrates that five genomically different phage, representing three genera, could be efficiently encapsulated (>95%) and remained viable (>87% recovery) following exposure to simulated gastric conditions (pH 2, 3.2 mg/mL pepsin, 37 °C for up to 1 h). Additionally, all phage were readily released from the microcapsules at pH 7.5 as well as after exposure to conditions mimicking the duodenum (pH 7, 10 mg/mL pancreatin, 37 °C).

The gastric acid barrier is a known challenge of oral phage therapy in livestock in general, and monogastric species in particular [61]. Residency time and pH in the GIT of livestock varies according to species and the feed/fasted state of each animal [61]. Real-time readings of pH using Heidelberg capsules show that the stomach and gizzard pH ranges from 0.79 to 3.64 for pigs, and 0.50 to 5.94 for poultry [62]. Proteins entering this acidic environment are often denatured and digested through a combination of hydrochloric acid and enzymes such as pepsin (optimally active at pH 2) [63]. Under these conditions, most tailed phage lose viability rapidly [17,64]. In the present study, the numbers of free (unencapsulated) phage were reduced to below detectable limits (10 PFU/mL) within 10 min of exposure to pH 2–2.5, suggesting that unprotected phage would be unlikely to successfully transit through the gastric acid barrier, even if the exposure time was short. It is noteworthy that phage RA140 had slightly higher reduction in titre (5.18 log_10_ PFU/mL) than all others tested here, and, more specifically, when compared to the other closely related Jerseyviruses in the panel (phage RA112 and RA124). However, this difference was not evident after encapsulation.

Encapsulation may protect phage to varying degrees against adverse environmental conditions, which could otherwise result in unpredictable or inconsistent therapeutic outcomes. The range of phage encapsulation efficiencies in the present study (95.2–98.6%) were consistent with EE% = 95.87 ± 2.12 reported for *Salmonella* phage SL01 [27]. Epseptimaviruses (e.g., RA40) and Jerseyviruses (e.g., RA112, RA124, and RA140) both have long, non-contractile tails, whereas Kutterviruses (e.g., RA148) possess contractile tails. Given that the EE% for these phage was not significantly different, it would appear that morphology was not a critical factor for encapsulation, at least for the examples used here. This suggests that cocktails of different *Salmonella* phage genera could be effectively co-encapsulated at similar efficiencies using the same vehicle, which would simplify product formulation and production. The inclusion of calcium carbonate in the formulation, while not significantly affecting EE%, resulted in particles which were significantly smaller. Other studies using ALG-CG formulations in variable proportions suggest a MPS ranging from 2–3 mm [27]. MPS may be influenced by several factors such as viscosity of the vehicle suspension or slower movement of the crosslink solution stirring. A previous study reported that when orally administering ALG beads (3.73 ± 0.04 mm) to chickens, no visible intact particles could be retrieved on the duodenum of dissected birds [65]. It is not yet known if smaller composite ALG-CG microcapsules could offer better delivery to the duodenum. This study is, to our knowledge, the first time the benefits of phage encapsulation in ALG-CG supplemented with CaCO_3_ have been demonstrated under simulated gastric conditions. Other authors that used ALG and CaCO_3_ (but no CG) to encapsulate *Salmonella* phage have found high EE% (>99%) [66], which is consistent with our study.

The protective effect of encapsulation varied according to phage genus and the formulation used. Encapsulation with ALG-CG alone offered some protection against incubation at pH 2 for 10 min (2–3 log_10_ PFU reduction, compared with >5 log_10_ PFU reduction in the unencapsulated control), but not for 60 min when phage recovery fell below detectable limits. ALG-CG encapsulation improved phage viability after both 10 min and 60 min incubation at pH 2.5 (1.5 log_10_ reduction compared with a 5 log_10_ PFU reduction in the control after 10 min), although recovery was less consistent after 60 min incubation. Given that GIT transit to reach the duodenum may range between 30 to 90 min in chickens [67], and more than 1.5 h in pigs [61], it is unlikely that the use of ALG-CG alone would allow therapeutically meaningful titres of phage to reach their bacterial targets in the small intestine and beyond.

The addition of CaCO_3_ to the ALG-CG formulation resulted in a significant increase (*p* < 0.05) in the recovery of all phage after incubation at both pH 2 and 2.5 up to 1 h, compared with both ALG-CG alone and unencapsulated phage. The improved phage viability in the current study formulation can be attributed to the slow gelation effect of alginate caused by adding CaCO_3_, which also during its dissolution allows diffusion of some CO_3_- ions into the medium to slightly increase the pH [49].

In the present study, we measured the pH of the SGF solution (10 mL) after incubation with the microcapsules (1 g) over the course of the experiment to determine whether initial pH conditions were maintained. After 1 h incubation at pH 2 and 2.5, the pH increased by 0.5 and 0.3, respectively, which was not sufficiently different from the initial conditions to explain the protective effect. The total amount of CaCO_3_ contained in 1 g of microcapsules is 10 mg and, by mixing 10 mg CaCO_3_ powder with 10 mL pH 2 SGF, the final pH measured was 8.50 ± 0.02. It is clear the effect of CaCO_3_ is largely confined to the microcapsule environment and is not due to the modification of the external pH environment. One potential advantage of using CaCO_3_ with ALG microcapsules is that the gas released on contact with acid may increase buoyancy, thereby reducing surface area exposure to gastric acid [48], while also creating a pH buffering environment within the microcapsules.

Previous phage therapy trials with poultry and pigs have used CaCO_3_ suspensions (∼30% *w*/*v*) administered via oral gavage to protect phage from the gastric environment [68]. Such disruption of the GI acid barrier, by forceful pH neutralization, is not only expensive, labourious, and impractical in a modern farm setting, but also exposes the animals to higher risk of infections caused by other pathogenic bacteria. Tennant et al. [69] demonstrated that the ID_50_ for hypochlorhydric mice could be reduced by between 0.5 and 1.85 log_10_ CFU for pathogens such as *Salmonella*, *Yersinia*, and *Clostridium*. Similar findings have been reported where the use of proton pump inhibitors in mice was associated with increased susceptibility to *Citrobacter rodentium* infections [70]. Interestingly, increased gastric pH was also associated with dysbiosis and metabolite composition in the small intestine, suggesting concomitant impacts in other areas of the intestinal tract. In modern farm settings, delivery of alginate microcapsules mixed in feed would be a practical option for the farmer. However, it is necessary to optimise the particles to be delivered in such conditions, e.g., dried after gelation, as some authors have demonstrated for alginate-based microcapsules [71]. The pH in the stomach and gizzard has been found to increase after feeding, up to 3.64 and 8 in pigs [61,62], and 5.94 in poultry [62]. These conditions may be sustained in pigs for an average of 2 h, although there is considerable variation at the individual level [61]. Water consumption also leads to increased gastric pH in pigs [61] and humans [72] but this tends to be short-lived (3 min) compared with the effect of feed. As such, the viability of phage delivered with feed, or around the time of feeding, may be improved compared with delivery via water. However, gastric retention time can also be prolonged after feeding which can delay microcapsule delivery and phage release into the duodenum. Optimising phage therapy in livestock will require consideration of factors such as feed particle size and composition on delivery, and how these can best be balanced with the nutritional requirements of the animals. 

## 5. Conclusions

In this study, we demonstrated that phage encapsulated in alginate–carrageenan-CaCO_3_ microcapsules offered excellent protection from a simulated gastrointestinal environment for phage representing three diverse genera. The inclusion of CaCO_3_ within the formulation, rather than delivery as a suspension by oral gavage, offers a cheaper and more effective method to protect therapeutic phage cocktails against the gastric acid barrier without increasing the risk of dysbiosis or infection with non-target pathogens.

## Figures and Tables

**Table 1:** DNA Sequencing and assembly statistics.

Phage	No. of Reads	No. of Subsampled Reads	Genome Size (bp)	× Coverage	Accession Number	Phage Genus
RA40	3.6 M	300 K	111,611	×26.47	OR242313	*Epseptimavirus*
RA112	4 M	20 K	42,100	×30.02	OR242314	*Jerseyvirus*
RA124	4.9 M	20 K	42,100	×29.9	OR242315	*Jerseyvirus*
RA140	3.7 M	20 K	42,022	×32.72	OR242316	*Jerseyvirus*
RA148	3.3 M	1 M	159,063	×29.02	OR242317	*Kuttervirus*

**Table 2:** Phage encapsulation efficiency (EE) and mean particle size (MPS). The mean percentage of phage which were retained in the microcapsules (±standard error, SE) is presented for sodium alginate and κ-carrageenan (ALG-CG) and for sodium alginate, κ-carrageenan, and calcium carbonate (ALG-CG-CA) formulations, based on three biological replicates. The mean particle size (±standard error, SE, *n* = 30) is displayed in millimeters (mm). Differences between MPS of ALG-CG and ALG-CG-CA groups were determined using an unpaired two-tailed *t*-test (* *p* ≤ 0.01, *** *p* ≤ 0.0001).

	EE % ± SE		MPS ± SE	
Phage	ALG-CG	ALG-CG-CA	ALG-CG	ALG-CG-CA
RA40	97.2 ± 0.84	97.0 ± 0.63	1.97 ± 0.02 ***	1.78 ± 0.02
RA112	98.5 ± 1.14	97.1 ± 1.28	1.90 ± 0.02 *	1.78 ± 0.03
RA124	98.2 ± 1.66	96.3 ± 0.83	1.94 ± 0.02 ***	1.81 ± 0.02
RA140	96.3 ± 1.30	98.6 ± 0.78	1.94 ± 0.02 ***	1.80 ± 0.03
RA148	95.2 ± 0.62	98.2 ± 0.81	1.92 ± 0.02 ***	1.80 ± 0.02

## Data Availability

Data supporting this study, and NCBI/Genbank accession numbers for genomes of the phage used, are included within the article.
